# Effect of long-term negative energy on appetite hormone levels in individuals with prediabetes and diabetes

**DOI:** 10.1590/1806-9282.20240897

**Published:** 2025-01-10

**Authors:** Gülşah Alyar, Fatma Zuhal Umudum, Neslihan Yüce, Nergis Akbaş

**Affiliations:** 1 Ataturk University, Vocational School of Health Services – Erzurum, Turkey.; 2 Ataturk University, Faculty of Medicine, Department of Medical Biochemistry, AD – Erzurum, Turkey.; 3 Yalova University, Faculty of Medicine, Department of Medical Biochemistry, AD – Yalova, Turkey.

**Keywords:** Appetite regulating hormone, Calorie restriction, Diabetes, Exercise, Prediabetes

## Abstract

**OBJECTIVE:**

Calorie restriction and exercise are commonly used first interventions to prevent the progression of prediabetes and alleviate the symptoms of type 2 diabetes. Our study was designed to determine the effect of the energy deficit caused by long-term (12-week) calorie restriction and exercise programs on appetite responses in obese individuals with prediabetes and type 2 diabetes.

**METHODS:**

Calorie restriction and exercise programs appropriate for age, gender, and work environment were applied to 22 individuals with prediabetes and 22 with type 2 diabetes participating in the study for a period of 12 weeks. Ghrelin, glucagon-like peptide-1, and peptide tyrosine tyrosine values of samples taken before and after treatment were determined by the enzyme-linked ιmmunosorbent assay method.

**RESULTS:**

Appetite hormone levels did not change after calorie restriction and exercise in the prediabetes group (p>0.05). In the diabetes group, calorie restriction and exercise significantly increased ghrelin and peptide tyrosine tyrosine concentrations (p<0.005). Additionally, when all patients were evaluated together, ghrelin, glucagon-like peptide-1 and peptide tyrosine tyrosine levels differed significantly after the intervention (p<0.005).

**CONCLUSION:**

The energy deficit created by long-term calorie restriction and exercise did not modulate the appetite hormones in prediabetic and obese individuals. However, increased ghrelin and peptide tyrosine tyrosine levels in individuals with diabetes support that the same treatment program is an effective method to regulate appetite hormones.

## INTRODUCTION

Type 2 diabetes (T2D) is a common metabolic disorder that shares common risk factors and comorbidities with obesity^1^. In the pathogenesis of T2D, metabolic dysfunction begins with insulin resistance as a result of impaired insulin secretion and continues with hyperinsulinemia and abnormal carbohydrate/lipid metabolism^2^. Following the disruption of carbohydrate metabolism, the pathological process progresses to prediabetes and diabetes. Although the blood glucose level in prediabetes mellitus (PDM) is higher than normal, it has not yet reached the accepted limit value for diabetes^3^. However, calorie restriction and exercise interventions are important approaches that offer the chance to stop and delay the progression of the disease, especially in the early stages^4^. Both applications have improving effects on blood glucose and insulin levels. Acute and long-term calorie restriction increases hepatic insulin sensitivity as well as improves β-cell function^5^. In addition, exercise reduces blood sugar levels by stimulating glucose uptake in peripheral tissues and reducing insulin resistance^6^. Additionally, the negative energy balance resulting from the combination of calorie restriction and exercise regulates appetite and relieves symptoms of diabetes by modulating hormones^7^. Regulation of appetite is achieved by the hypothalamus integrating signal molecules sent to peripheral organs through direct and indirect means. Ghrelin, peptide tyrosine tyrosine (PYY), and glucagon-like peptide-1 (GLP-1) are gastrointestinal peptides that play an important role in ensuring this integration^8^. A recent study found that 2 weeks of exercise did not affect the basal and postprandial ghrelin levels of adults with prediabetes but increased GLP-1 levels^9^. In the literature review on the subject, no research was found examining the effects of long-term negative energy deficits on appetite hormones in this population. In this context, our study primarily examined the appetite hormone levels of obese individuals with prediabetes and diabetes. Then the effect of the energy deficit created by long-term (12-week) calorie restriction and exercise program on appetite hormone levels was determined.

## METHODS

The study, which was approved by the Atatürk University Clinical Research Ethics Committee’s decision numbered B.30.2.ATA.0.01.00/29, was carried out in the laboratory of Atatürk University Faculty of Medicine, Department of Medical Biochemistry. The study consisted of individuals who applied to the Obesity Center between September 2022 and December 2022. The volunteers were informed about the study, read the “Informed Voluntary Consent Form,” and were included in the study after obtaining approval. Fasting serum samples were collected from participants after written informed consent was obtained. Adults aged 18–65 years with body mass index (BMI) ≥30 kg/m^2^, diagnosed with diabetes mellitus or prediabetes, were included in the study. Exclusion criteria include having a chronic disease other than obesity and using medications that affect the appetite mechanism. A calorie restriction and exercise program was applied on a voluntary basis to obese individuals with PDM and diabetes mellitus (DM) who applied to the Obesity Center in a province in Turkey. In our study, a calorie restriction (1,000–1,500 kcal/day) and exercise (at least 5,000 steps/day) program suitable for age, gender, and BMI parameters was created for the case group. The values of appetite hormones in serum samples of the case group were analyzed by the enzyme-linked ιmmunosorbent assay (ELISA) method and commercial kit (SunRed) by taking venous blood samples before and 12 weeks after starting the calorie restriction and exercise program.

### Statistics

The data obtained from the study were analyzed using the Analyse-it Software (Analyse-it Software, Ltd., Leeds, United Kingdom) and the Statistical Package for the Social Sciences (SPSS) 23 package program. The suitability of the data distribution to normal distribution was evaluated with the Kolmogorov-Smirnov test. Student’s t-test, one of the parametric tests, was used for normally distributed data. The Kruskal-Wallis test was used for multiple group comparisons. Paired samples t-test and Wilcoxon test were used to analyze the data of the dependent groups. The statistical significance level was based on p<0.05.

## RESULTS

The average age of 44 individuals—22 with PDM and 22 with DM—who participated in the study was 48 years. While the average weight value of the PDM group before treatment was 101.5, this value decreased to 95.5 after treatment. While the average weight value in the DM group was 108.5 before treatment, it decreased to 99.7 after treatment. The average BMI value is 40.5 and the average waist circumference value is 110.42. The BMI of the DM group is 42.6 and the average waist circumference is 118.5.

Appetite hormone levels of the PDM group before and after combined treatment are given in Table 1. In this group, although basal ghrelin, GLP 1, and PYY levels increased after treatment compared to before treatment, they were not significant.

**Table 1.** Appetite hormone levels before treatment and after treatment in the prediabetes mellitus.

	PDM (before treatment)	PDM (after treatment)	p-value
Ghrelin (pg/mL)	2331.05±625.76	2590.16±639.39	0.158
PYY (pg/mL)	112.81±26.07	116.85±18.62	0.268
GLP-1 (pmol/L)	124.65±40.48	126.20±40.61	0.895

p<0.05. PDM: prediabetes mellitus.

Appetite hormone levels of the DM group before and after combined treatment are shown in Table 2.

**Table 2.** Appetite hormone levels before and after treatment in the diabetes mellitus.

	DM (before treatment)	DM (after treatment)	p-value
Ghrelin (pg/mL)	2330.99±1228.49	2653.17±1236.96	0.026*
PYY (pg/mL)	112.36±43.06	126.71±33.38	0.044*
GLP-1 (pmol/L)	113.80±48.57	107.12±39.20	0.428

*p<0.05. DM: diabetes mellitus. “*” indicates that the basal ghrelin and PYY levels in the DM group increased significantly after treatment.

In the DM group, basal ghrelin and PYY levels increased significantly after treatment. Although GLP-1 levels decreased, there was no significant change.

Appetite hormone levels of all patients participating in the study before and after treatment are shown in Table 3. Ghrelin and PYY levels of all participants increased significantly after treatment.

**Table 3.** Appetite hormone levels before and after treatment of all patients.

	All patients (before treatment)	All patients (after treatment)	p-value
Ghrelin (pg/mL)	2331.02±963.47	2621.67±973.61	0.011*
PYY (pg/mL)	112.58±35.18	121.78±27.17	0.021*
GLP-1 (pmol/L)	119.23±44.52	116.66±40.61	0.716

*p<0.05. “*” indicates that the basal ghrelin and PYY levels in the DM group increased significantly after treatment.

## DISCUSSION

This study was designed to evaluate the effects of 12-week energy intake limitation and physical movement on ghrelin, GLP-1, and PYY levels in PDM and T2D individuals. As a result of the study, it was observed that long-term energy deficit improved appetite regulation in the DM group without compensatory responses. Calorie restriction and physical movement are effective approaches that prevent the progression of prediabetes in the early stages and relieve diabetes symptoms^10^. Restricting food intake provides improved β-cell function and insulin sensitivity of peripheral tissues^3^. Additionally, physical activity that increases energy expenditure relieves pancreatic β cells from metabolic stress induced by excess energy^5^. Physical movement improves the glycemic index by allowing muscles to take up glucose independently of insulin^1^. Another notable result of the improvement resulting from these interventions is the positive change in appetite hormones that occurs with weight loss^8^. In our study, ghrelin, GLP-1, and PYY levels were determined to be low in the PDM group before treatment. Appetite hormone levels did not change after the 12-week treatment program applied to PDM individuals. A recent study reported that short-term (2-week) exercise did not affect basal and postprandial ghrelin levels in adults with prediabetes but increased GLP-1 levels^9^. Similarly, Malin et al. stated that 2 weeks of exercise increased GLP-1 release and increased pancreatic beta cell function in obese people with prediabetes^11^. Another important finding of our study is that after 12 weeks of combined treatment applied to diabetic obese patients, ghrelin and PYY levels increased significantly, while GLP-1 levels did not change. Ghrelin, an orexigenic hormone, is also effective in regulating glucose and lipid metabolism. Decreased ghrelin levels in obesity have been associated with increased insulin levels^12^. The satiety hormone GLP-1 increases insulin secretion and cell proliferation. Decreased satiety hormone levels in obese individuals trigger the development of T2D^13^. In our study, pretreatment ghrelin, GLP-1, and PYY levels of obese diabetic individuals were determined to be low, consistent with previous studies. This result shows that calorie restriction and physical activity are sensitive to appetite responses. However, appetite hormone levels, which were also low in the prediabetes group, were not different between the diabetes group. Data from studies investigating the effects of acute and long-term calorie restriction and exercise on appetite hormone levels are very variable^14^,^15^. Research has shown a wide range of results, from no change in appetite perceptions to an increase/decrease in hunger hormones and an increase/decrease in satiety hormones^16^,^17^. However, studies were conducted on both normal-weight and obese people. It is thought that this inconsistency in the findings is due to sample diversity and methodological differences in the studies^18^. For this reason, studies focusing specifically on the population are needed.

## CONCLUSION

The energy deficit caused by long-term calorie restriction and exercise did not modulate the appetite hormones of prediabetic obese people. However, the same treatment results show that it is an effective method for regulating appetite hormones in obese people with diabetes.
